# AMPed up to treat prostate cancer: novel AMPK activators emerge for cancer therapy

**DOI:** 10.1002/emmm.201303737

**Published:** 2014-02-21

**Authors:** Matthew J Schiewer, Karen E Knudsen

**Affiliations:** 1 Department of Cancer Biology, Thomas Jefferson UniversityPhiladelphia, PA, USA; 2 Kimmel Cancer Center, Thomas Jefferson UniversityPhiladelphia, PA, USA; 3 Department of Urology, Thomas Jefferson UniversityPhiladelphia, PA, USA; 4 Department of Radiation Oncology, Thomas Jefferson UniversityPhiladelphia, PA, USA

## Abstract

Despite recent advances in the treatment for metastatic prostatic adenocarcinoma, clinical management of this tumor type remains a major challenge, and there is as of yet no durable cure for advanced disease. Developing pathways that could be co-targeted alongside the androgen receptor or that would otherwise thwart the development of the CRPC is a current translational and clinical priority. In this issue, a new study by Zadra *et al* identifies the energy sensor AMPK (5′ AMP-activated kinase) as a viable therapeutic target in prostate cancer.

Despite recent advances in the treatment for metastatic prostatic adenocarcinoma (MacVicar & Hussain, 2013), clinical management of this tumor type remains a major challenge, and there is as of yet no durable cure for advanced disease. Prostate cancer generally responds poorly to standard chemotherapy, but is heavily dependent on signaling of the androgen receptor (AR) for growth and survival (Knudsen & Penning, 2010). Thus, the mainstay of treatment targets this dependence, combining mechanisms to either deplete the AR of ligand or through the use of direct AR antagonists. Although these strategies are initially effective, recurrent tumors deemed “castrate-resistant prostate cancer” (CRPC) ultimately arise. Developing pathways that could be co-targeted alongside AR or that would otherwise thwart the development of the CRPC is a current translational and clinical priority.

In this issue, a new study by Zadra *et al* (2014) identifies the energy sensor AMPK (5′AMP-activated kinase) as a viable therapeutic target in prostate cancer. AMPK is a serine/threonine kinase that functions as a metabolic sensor that is sensitive to AMP/ATP levels and serves to enhance ATP generation (Hardie, 2011). In mammalian cells, the kinase exists as a heterotrimer comprised of a single α (catalytic) subunit in addition to two ß and γ regulatory subunits— as such, variant AMPK complexes exist and may be divergent dependent on cellular context. In spite of this complexity, common activation events occur when the subunit is phosphorylated on threonine 172, which occurs in the T-loop(Carling *et al*, 2012).

Developing pathways that could be co-targeted alongside AR or that would otherwise thwart the development of the CRPC is a current translational and clinical priority.

The cellular function of AMPK is to respond to metabolic state and oncogenic stress. Activated AMPK induces catabolic metabolism and suppresses the anabolic state, thereby inhibiting cellular proliferation and potentially serving a tumor suppressive role (Liang & Mills, 2013). Consistent with this idea, AMPK loss can promote tumor progression, as genetic deletion of the AMPK α1 subunit potentiated Myc-induced lymphomagenesis (Faubert *et al*, 2013). At the molecular level, the tumor suppressive role of activated AMPK is associated with inhibition of cell cycle progression, cholesterol and fatty acid synthesis, and mTORC1 signaling, in addition to promotion of cell death (via both apoptosis and autophagy) (Fogarty & Hardie, 2010). Given these properties, there is an intensive effort toward identification of means to directly activate AMPK.

The anti-diabetic compound metformin provides a known clinical means to induce metabolic stress and is currently under assessment in clinical trials (Pierotti *et al*, 2013). However, this agent does not directly target AMPK or the upstream kinase LKB1(Hardie, 2006) and has not shown consistently promising results in the context of prostate cancer(Margel *et al*, 2013). By contrast, Zadra *et al* (2014) took advantage of a novel small molecule activator of AMPK and challenged a putative anti-tumor effect of this compound using a series of *in vitro* and *in vivo* models. This new agent, MT 63–78 (also called Debio 0930), was identified by a screen using a recombinant AMPK complex (α1 ß1 γ1), wherein it was shown using cell-free systems that MT 63–78 not only activates AMPK in a dose-dependent manner, but inhibits dephosphorylation of AMPK on threonine 172. Further functional analyses revealed that MT 63–78 acts primarily by binding to the ß1 subunit, indicating that this small molecule activator is likely specific to the ß1 containing class of AMPK complexes.

Although the compound showed anti-tumor effects in a number of different cancer cell types, MT 63–78 exerted robust growth-inhibitory effects in AR-positive prostate cancer cells that are sensitive to AR-targeted therapeutics, as well as in a number of model systems reflective of castrate-resistant disease. The cellular response to MT 63–78 included G2/M cell cycle arrest — such cell cycle inhibition was sustained in cells deficient in p53 function, suggesting a p53-independent effect. This is of interest, since AMPK is known to regulate p53 post-translational modification and to modify the action of this tumor suppressor, and p53 deregulation is over-represented in PCa. Sustained activation of AMPK by MT 63–78 ultimately resulted in activation of the intrinsic apoptotic cell death pathway that was attributed to a block on correct cytokinesis, similar to effects previously attributed to metformin. However, unlike metformin, the biological effects of MT 63–78 required functional AMPK, thus demonstrating specificity.

Strikingly, molecular investigation revealed that the growth-inhibitory effects of MT 63–78 could not be attributed to metabolic stress or suppression of the mTORC1 pathway, as might be expected. Rather, the underlying mechanism of action was elegantly linked to blockade of *de novo* lipogenesis. Treatment of prostate cancer cells with MT 63–78 resulted in a dose-dependent reduction in *de novo* phospholipid production and a marked decrease in the generation of neutral lipids (including triacylglycerides, diacylglycerols, cholesterol, and cholesterol esters) that was associated with growth suppression. Causation was demonstrated, in that addition of palmitate or mevalonate (products of 3-hydroxy-3-methylglutaryl-CoA reductase and fatty acid synthase, FASN) could partially restore prostate cancer cell growth even in the presence of MT 63–78, while no effect was seen in cells untreated with MT 63–78. These findings strongly support the contention that the growth-inhibitory functions of AMPK are mediated largely via suppression of cholesterol and fatty acid synthesis. Importantly, MT 63–78 outperformed the commercially available AMPK activator A-769662 in every tested capacity.

The preclinical impact of MT 63–78-mediated AMPK activation and cancer cell growth suppression was further explored with an eye toward development of the agent as a means to treat advanced prostate cancer. Justification is notable, as it is well appreciated that enhanced lipogenesis is a hallmark of advanced prostate cancer (Swinnen *et al*, 2004; Massie *et al*, 2011). Remarkably, MT 63–78 was shown to robustly enhance the anti-tumor effects of two AR antagonists that are utilized clinically (bicalutamide and enzalutamide), and this cooperative effect was observed both in cells that are sensitive to AR inhibition alone, as well as in CRPC models that are refractory to the AR antagonists as single agents. While the underlying mechanisms remain undetermined, it was tantalizingly observed that MT 63–78 reduces both AR levels and AR activity when combined with the AR antagonists *in vitro*. Parallel *in vivo* analyses also support the concept that AMPK activation could result in significant anti-tumor effects. Significant reduction in tumor growth was observed upon MT 63–78 treatment using a human tumor xenograft model of AR-positive disease. Consonant with the molecular analyses, MT 63–78-mediated AMPK activation was associated with suppressed lipogenesis, inhibition of mTORC1 pathways, and enhanced apoptosis. Host animals demonstrated no significant change with regard to body weight, serum glucose levels, or triglyceride levels when maintained on a normal chow diet. Taken together, these studies reveal promising effects of AMPK activators as novel anti-prostate cancer agents.

Finally, genetically engineered mouse models were utilized to assess the role of AMPK catalytic activity in prostate cancer development. This was critical, as there is some uncertainty as to whether AMPK serves solely as a tumor suppressor or can function as a context-dependent oncogene(Liang & Mills, 2013). To address this, transgenic mice engineered to harbor prostate-specific FASN expression (FASN-Tg) were crossed to animals defective in AMPK. FASN-Tg animals are known to develop age-dependent prostatic epithelial neoplasia (PIN); however, loss of the α2 subunit of AMPK resulted in increased PIN formation, suggesting for the first time that loss of AMPK α2-associated catalytic activity is sufficient to promote FASN-induced tumorigenic phenotypes. These genetic studies further demonstrate that activation of AMPK has a significant impact on PCa biology.

On balance, this study provides novel insight into the role of AMPK in suppressing tumor formation and/or growth, and credentials AMPK activators as a novel means to treat advanced, intractable prostate cancer. The elegant molecular and *in vivo* investigations provide robust evidence that AMPK activation attenuates tumor growth primarily through suppression of lipogenesis, thus putting forth the provocative hypothesis that AMPK-activating compounds may be particularly effective in tumors with heightened lipogenic activity. Given the role of increased lipid and cholesterol biosynthesis in the generation of castration resistance, it is clear that prostate cancer may be a suitable tumor type for AMPK-directed intervention. However, some key questions remain. First, what biomarkers can be used to identify tumors that are responsive to AMPK activators? Delineation of such will be imperative for determining which patient(s) might most benefit from AMPK-directed agents. Second, the observation that AMPK-activating compounds can act in concert with AR antagonists is enticing, but the underlying mechanisms remain unsolved. Elucidating this process may be critical for development of AMPK activators as therapeutics to treat advanced prostate cancer, given the multitude of mechanisms by which AR is reactivated after hormone therapy. Finally, mechanisms underpinning specificity of AMPK activity should be addressed, in the light of data implicating AMPK as a context-specific tumor suppressor and tumor promoter. Despite these remaining questions, the present study clearly illuminates the potential for utilizing AMPK activators as therapeutic agents and “amps up” enthusiasm for clinical development (Fig 1).

**Figure 1:**
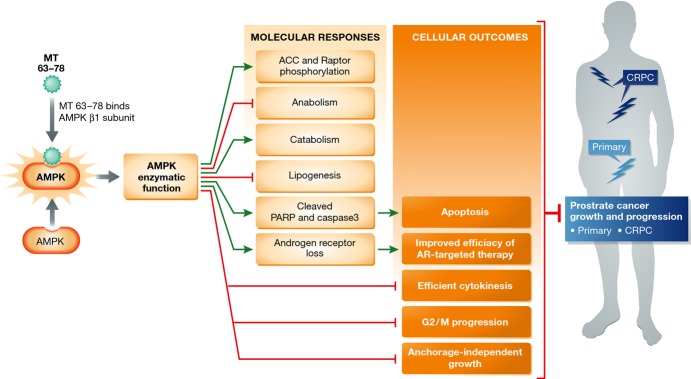
MT 63–78 (green burst) binds to the β1 subunit of AMPK, activating its enzymatic function. Activation of AMPK results in multiple molecular (light orange) and cellular (dark orange) effects. The overall result of AMPK activation is reduced prostate cancer growth in models of hormone-therapy-sensitive and castration-resistant disease. Green arrows indicate positive regulation, and red squared lines indicate negative regulation. Light blue star on silhouette indicates primary prostate cancer, and dark blue stars indicate metastatic and castration-resistant prostate cancer (CRPC).

… this study provides novel insight into the role of AMPK in suppressing tumor formation and/or growth and credentials AMPK activators as a novel means to treat advanced, intractable prostate cancer.
